# User Input in the Development of Digital Sexual Health Tools: A Scoping Review and Guidance for Tool Developers

**DOI:** 10.1111/hex.70360

**Published:** 2025-07-28

**Authors:** Alicia King, Ethan Cardwell, Eric Chow, Paul Flowers, Mark Gilbert, Kath Albury

**Affiliations:** ^1^ School of Translational Medicine Monash University Melbourne Victoria Australia; ^2^ Melbourne Sexual Health Centre Alfred Health Melbourne Victoria Australia; ^3^ Melbourne School of Population and Global Health The University of Melbourne Melbourne Victoria Australia; ^4^ Psychological Sciences and Health University of Strathclyde Glasgow Scotland United Kingdom; ^5^ School of Population and Public Health The University of British Columbia Vancouver British Columbia Canada; ^6^ School of Social Sciences, Media, Film and Education Swinburne University of Technology Melbourne Victoria Australia

**Keywords:** digital Health, health information seeking, mobile applications, patient participation, sexual health, sexually transmitted diseases

## Abstract

**Background or Context:**

Studies reporting the use of digital tools to promote the prevention and treatment of sexually transmitted and blood borne infections (STBBIs) have proliferated in recent years. Previous reviews highlight variability in the input sought from users in tool development, and its contribution to impact.

**Objective:**

This scoping review sought to describe approaches to seeking and utilising user input, with the goal of providing guidance for developers.

**Search Strategy:**

Searches were conducted in MEDLINE, PsycInfo, and the Social Science Citation Index and results screened by two reviewers. The reference lists of included studies and review papers were also checked.

**Inclusion Criteria:**

Peer reviewed qualitative and mixed methods studies seeking user input on digital tools promoting the prevention and treatment of STBBIs, from prototyping onwards, published from after 2014 in English, were included.

**Data Extraction and Synthesis:**

Reported methods and findings were charted in Excel and synthesised using content analysis to provide an overview of methods and domains of user input and utilisation of this input.

**Main Results:**

A total of 1838 unique titles and abstracts and the full text of 50 publications were screened. Data was charted from 37 eligible studies reporting findings from 34 projects developing digital health tools, including smartphone/tablet applications, websites/web‐based applications, chatbots, interactive automated SMS, and purpose‐built tools within dating and social media applications. Studies reported on tools developed for use by diverse target populations. The most common domain of input reported was usability (*n* = 31), while others—namely, satisfaction (*n* = 27), acceptability (*n* = 25), formative (*n* = 24), impact (*n* = 22), accessibility (*n* = 17), and engagement (*n* = 11)—were reported less consistently. User views were sought using qualitative methods such as interviews, focus groups and open‐ended survey questions, more often in combination with quantitative measures such as participant‐rated measures and engagement analytics. User suggestions for changes were reported in relation to three in four projects studied but incorporation of changes in less than half of projects.

**Discussion and Conclusions:**

This review demonstrates considerable homogeneity in reported user input in the development of digital health tools. Input from users as co‐designers may improve the impact of tools on their intended outcomes.

**Patient and Public Contribution:**

This literature review brought together a group of researchers who have sought user input in the development of digital sexual health tools, but, due to resource limitations, did not involve potential users themselves, who are of diverse and disparate groups.

## 1 Introduction

Digital tools to promote the prevention and treatment of sexually transmitted and blood‐borne infections (hereafter referred to as ‘digital sexual health tools’) are a growing part of the sexual health landscape. Examples include sexual and reproductive health (SRH) education platforms, self‐testing websites, smartphone applications and, more recently, AI‐powered chatbots and photo diagnosis applications. Their value in impacting their intended outcomes, however, has yet to be conclusively proven. In the past decade, no less than six systematic and scoping reviews on digital sexual health (DSH) tools have been published [1, 2, 3, 4, 5, 6]. Almost universally, they report mixed or ‘preliminary’ results [1, 2, 4] and even the potential to produce outcomes counter to their purpose [3]. Limitations in the design of research into DSH tools have been identified, particularly the rigour of evaluations [2, 6], but also the variability in seeking and incorporating user input (i.e., perspectives, suggestions and feedback from potential users) during the design process.

Reviews of DSH tools have highlighted inadequate tailoring of tools to meet the needs of users as a potential barrier to their effectiveness [2, 5]. Seeking user input may result in the translation of tool effectiveness to real world impact by supporting uptake and repeated use [7]. Veronese et al. [1] found that DSH technologies that benefited from user input in the design process had a greater impact. Differences in approaches to seeking user input across the technology and public health sectors are evidenced in the heterogenous methods used in tool development and evaluation [8]. While patient and public involvement, including co‐design, is increasingly recommended in the development of health products and services, there are few studies linking processes undertaken to seek user input with outcomes [9, 10]. With reference to DSH tools, Balaji et al. [4] identified the need for additional user experience constructs beyond ‘current technology acceptance models’. Building on these findings, this review sought to take a broader scope on the DSH literature to explore how user input in the design of tools might be improved.

In this review, we describe the methods used in the development of DSH tools to seek input from end users of tools, the domains of input reported, and evidence of their incorporation into tool design. Consistent with these aims, we chose a scoping review method to map qualitative and mixed methods research studies of DSH tools and answer the following questions: *(1) What approaches have been used to seek the views of potential users of DSH tools? (2) To what extent does the reporting of user input in publications suggest their input is meaningfully shaping the development of DSH tools?*.

## 2 Methods

This review followed the process described by Arksey and O'Malley [11] and further refined by O'Brien, Colquhoun [12]. It is reported with reference to the Preferred Reporting Items for Systematic reviews and Meta‐Analyses extension for Scoping Reviews (PRISMA‐ScR) [13]. No protocol was published, and the review was not registered.

### 2.1 Eligibility Criteria

Consistent with our focus on user input we limited our review to qualitative or mixed methods studies that sought user views on purpose‐built DSH tools, including early prototypes of any fidelity (e.g., paper mock ups, wireframes) through to pilot studies of developed tools. After an initial search, we opted to limit our search to the previous 10 years as few tools, except websites, were reported before 2015. Studies conducted before prototyping (i.e., in the formative stages of development) were not included, as we were interested in exploring the use of user input over time, but mention of earlier studies in later studies was captured in data charting. We chose to exclude serious video games from our review as the gamification of health interventions requires a methodological approach that is quite different from other digital health interventions, and an evidence map was recently published of these interventions [14]. We also chose to exclude digital vending machines as descriptions we found in published studies did not meet our criterion of providing information and services tailored to user inputs. As described by Arksey and O'Malley [11], the focus of our study was iteratively refined as we developed an understanding of the breadth of research in the field, resulting in the final inclusion and exclusion criteria shown in Table 1, that were applied during study selection.

**Table 1:** Inclusion and exclusion criteria for study selection.

Inclusion criteria	Exclusion criteria
Studies that sought user input on tools to promote the prevention and treatment of sexually transmitted and blood borne viruses by providing automated responses that vary based on user inputs (including prototypes of any fidelity) Qualitative or mixed methods studies Published from 2015 onwards Published in English	Studies conducted before prototyping Clinicians or caregivers as only research participants Use of existing platforms to promote sexual health outcomes Use of tool to support ongoing engagement with care or treatment adherence of people living with HIV. Tools for use in a clinical or educational setting, except where supporting access for users without a device/internet/private space for use Studies of serious video or smartphone games and digital vending machines Grey literature (e.g., unpublished work, conference abstracts, pre‐prints of manuscripts undergoing peer review)

### 2.2 Information Sources

Peer reviewed publications were sourced from database searches in MEDLINE (Ovid), PsycInfo (Ovid), and the Social Science Citation Index (Web of Science). At the time of searching, auto alerts for all three searches were created and checked until 12 September 2024 and relevant studies subjected to the same screening process described below. No grey literature was included.

### 2.3 Search

Search terms were chosen to capture each of four key concepts in the review questions. Terms were searched as identical key words in all databases and similar related subject headings specific to each database. An example of the terms used in MEDLINE is provided in Table 2. Key word and subject headings for each concept were combined using the ‘or’ Boolean operator then the search results for each concepts combined using ‘and’ (Files [Link], [Link], [Link]).

**Table 2:** Search terms used in MEDLINE.

Digital health tools	STIs (including HIV)	Sexual health care and prevention	User views
Digital health^a^^,^ b Mobile applications^a^^,^ b User‐Computer Interface^b^	Sexually Transmitted Diseases^a^^,^ b Sexually Transmitted Infections^a^ Sexual Transmissible Infections^a^ Chlamydia^a^ Infections^b^ Gonorrhea^a^ Gonorrhoea^a^ HIV^a^ Syphilis^a^,^b^ Herpes^a^ Simplex^b^ HPV^a^ Papillomavirus Infections^b^	Sexual health^a^^,^ b Sex Education^a^^,^ b Sexual behav*^a^^,^ b Healthcare engagement^a^ Healthcare seeking^a^ ‘Patient Acceptance of Health Care’^b^ Prevention^a^ Primary Prevention^b^ Secondary Prevention^b^ Condoms^a^^,^ b Self‐testing^a^^,^ b HIV Testing^a^^,^ b PrEP^a^^,^ b Pre‐Exposure Prophylaxis^b^ Anti‐HIV Agents^b^ Vaccinat*^a^^,^ b	Qualitative Research^a^^,^ b User experience^a^ Patient Participation^a^^,^ b Mixed method*^a^^,^ b ‘Survey^a^ s and Questionnaires’^b^ Codesign^a^ Human Centred Design^a^ Human Centred Design^a^^,^ b Universal Design^b^ Human computer interaction^a^ Participatory research^a^ Community‐Based Participatory Research^b^

^a^ Key word.

^b^ Subject heading.

### 2.4 Selection of Sources

Search results were uploaded to Covidence systematic review software (Veritas Health Innovation). Duplicates were removed by the automated function of this application and manually during screening, by sorting the uploaded citations by author and marking duplicates not identified by Covidence. All titles and abstracts were screened by A.J.K. and E.T.C. and conflicts resolved in discussion with K.A. The same process was applied at full‐text review. Additional studies were identified for screening by checking the references lists of studies included following full text review and any review papers found in our search results. Potential references found through citation searching were reviewed by A.J.K. and E.T.C. and any conflicts resolved by K.A.

### 2.5 Data Charting Process

Data charting was conducted in Excel. A.J.K. and E.T.C. each extracted data from half of the included studies by copying relevant text verbatim into predefined fields of a shared sheet. Fields of interest were drafted by A.J.K. and E.T.C. and refined in discussion with K.A. and P.F. A.J.K. and E.T.C. checked a random selection of the other's allocated studies and met to discuss differences in their extraction approach.

#### 2.5.1 Data Items

The fields used for data charting are provided in File S4. For most items, verbatim text was input into the field with the following exceptions. Where not clearly stated in the description of the tool, the purpose was determined based on the introduction of the paper. Where the research aim was not clearly stated, the domains of user input captured by research tools were entered (e.g., usability if System Usability Scale used).

### 2.6 Approach to Synthesising Results

To answer our research questions, we reviewed the data extracted from the included studies and applied the following methods of data synthesis.

#### 2.6.1 Domains of User Input Explored in Studies

A.J.K. and E.T.C. reviewed the data extracted from the *Research details* and *User input* fields in File S4 with reference to the following questions describing seven domains of user input. The questions were developed following familiarisation with the data set during charting and revised in consultation with co‐authors working in the DSH field (K.A., M.G., P.F.).

- Were potential users invited to make a formative contribution to design and scoping, before prototype development? (defined in this paper as *formative* input).
- Were potential users asked if they would be willing to use it or if they would prefer it to alternatives? (defined as *acceptability*)Were users asked about aspects of the tools that might prevent them or others from using the tool due to individual factors (e.g., low literacy, physical, sensory or cognitive impairment, English language proficiency)? (defined as *accessibility*).
- Were users asked how easy‐to‐use or user friendly the tool was? (defined as *usability*).
- Were users asked about their satisfaction or experience of using the tool at any stage (including if they would recommend it to others)? (defined as *satisfaction*).
- Did the study measure users’ spontaneous and/or in situ use of the tool or features of the tool beyond user testing (i.e., as the need for sexual health information or advice arose)? (defined as *engagement*).
- Were participants asked what impact the tool had or might have on the intended outcome (e.g., knowledge, healthcare seeking, HIV/STI testing, condom use)? (defined as *impact*).

**Figure 1:**
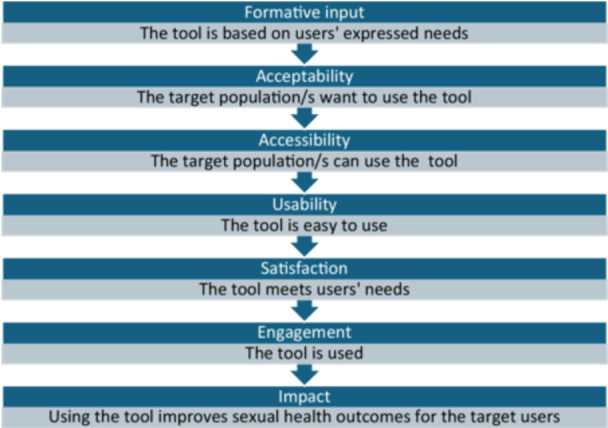
Domains of user input and their potential role in achieving impact.

A.J.K. and E.T.C. reviewed the stated aims and reported findings of each of the included studies and made a qualitative decision as to which domains were explored within the project, guided by the questions shown for each domain. Figure 1 depicts the domains of input reviewed in our data synthesis in a provisional theory of change [15] suggesting the potential role each type of input may have in achieving a tool's intended impact.

#### 2.6.2 Reporting of Impact of User Input on Tool Development

The impact of user input on the development and refinement of tools was synthesised by reviewing data extracted from individual studies in relation to:

- Participant *suggestions* for changes or additional features.
- Planned or executed *changes* to the tool in response to user input.

Examples of participant suggestions being incorporated into tools are reported in the results that follow.

## 3 Results

### 3.1 Selection of Sources of Evidence

**Figure 2:**
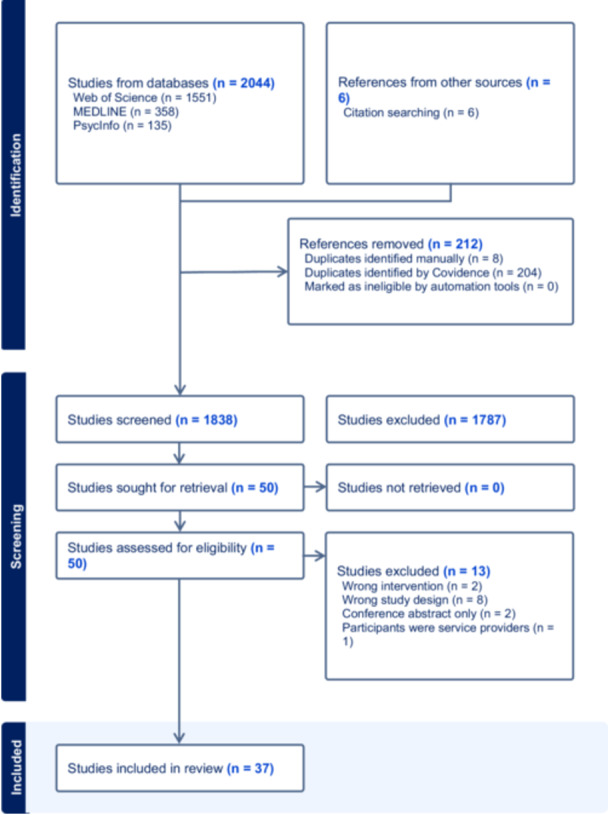
PRISMA flow diagram showing the process of study selection.

The process of study selection and the number of sources screened for inclusion is shown in Figure 2. In brief, 1838 unique titles and abstracts were screened, included three studies found through auto alerts after our initial searches and six from the reference lists of included studies and reviews. Fifty peer‐reviewed publications were subjected to full text review resulting 37 included studies.

### 3.2 Description of Included Studies

An overview of projects (*n* = 34) described in included studies (*n* = 37) is provided in Table 3. Six studies related to different phases of the same three projects, so the findings of these studies are reported together in the tables below. Thus, the attributes of 37 individual studies are reported in relation to 34 projects using individual DSH tools (*n* = 31) or multiple tools in combination (*n* = 3).

**Table 3:** Description of projects evaluating digital sexual health tools (*n* = 34).

First author/s and year	Name of tool/s	Country	Type of tool/s used	Purpose of tool/s	Tool features and functions	Target population
Adeagbo, 2021 [16]	EPIC‐HIV1 (Empowering People through Informed Choices for HIV)	South Africa	Smartphone or tablet application	HIV^a^ testing promotion	Informed consent info presented in story about character chosen by user.	Male youth and men
Anderson, 2022 [17]	MyPEEPS	USA	Smartphone or tablet application	HIV/STI^b^ education	Educational content.	Transmasculine adolescents GBMSM^c^
Bailey, 2016 [18]	The Men's Safer Sex website	UK	Website or web‐based application	Promotion of condom use	Interactive educational content.	Adult men who have sex with women
Biello, 2021 [19]	LYNX and MyChoices	USA	Smartphone or tablet application	HIV/STI self‐testing, PrEP^d^ promotion	Ordering and delivery of HIV/STI self‐test kits and information on PrEP.	Adolescent and youth MSM^e^
Braddock, 2023 [20]	PrEPBot	USA	Chatbot	HIV self‐testing, PrEP access	AI ‘TelePrEP’ navigator guiding users through online PrEP prescription and delivery.	Sexual and gender minority adolescents and young adults
Chandler, 2020 [21]; Chandler, 2023 [22]	SavvyHER (Sexual/HIV Health Electronic Empowerment Resource)	USA	Smartphone or tablet application	HIV education, service navigation, STI self‐testing. peer support	Health tracking; HIV testing and PrEP clinic locator; STI self‐test, condom and sexual pleasure item ordering; multimedia resources; peer support.	PrEP eligible Black/African American women
Cheah, 2024 [23]	Haris (chatbot) and MYHIV365 (website)	Malaysia	Chatbot	HIV education, mental health screening, service navigation	HIV self‐test ordering; MSM‐friendly clinic information; screening for depression.	MSM
Cordova, 2018 [24]	Storytelling 4 empowerment (S4E)	USA	Smartphone or tablet application	Promotion of condom use, HIV/STI and drug use education	Educational content delivered in storytelling in format.	Adolescents and youths
Danielson, 2016 [25]	Sistas Informing Healing Living Empowering (SiHLE Web)	USA	Website or web‐based application	HIV education	Interactive educational content.	African American adolescent girls
Engen, 2021 [26]; Baraitser, 2023 [27]	SH:24	UK	Website or web‐based application	Diagnosis and treatment of herpes and genital warts	Triage and photo diagnosis of lesions by a sexual health clinician; postal delivery of treatment; referral to in person care.	Not specified
Fitzpatrick, 2023 [28]	PrEP EmERGE	UK	Smartphone or tablet application	PrEP management and adherence	Access to results, medication reminders, vaccination history, upcoming appointments and one way (clinic‐ user) messaging.	PrEP users
Gilbert, 2017 [29]	GetCheckedOnline	Canada	Website or web‐based application	HIV/STI self‐testing	Risk assessment; recommendation of tests; printable pathology request.	Not specified
Goldenberg, 2015 [30]	Not reported	USA	Smartphone or tablet application	HIV education, HIV testing promotion, service navigation and self‐monitoring.	Personalised profile; HIV testing plan; HIV testing locator; event tracker for sexual encounters and HIV testing; HIV prevention tips; interaction credits for donation to HIV/LGBT^f^ organisations.	MSM
Greene, 2016 [31]	Keep It Up! (KIU!)	USA	Website or web‐based application	HIV education	Educational modules delivered in multimedia format (e.g., videos, animation, and games).	HIV‐negative, ethnically and racially diverse young MSM
Hughes, 2021 [32]	Nurx	USA	Website or web‐based application	PrEP access	Counselling, prescription and home‐delivery of medication.	People wishing to access PrEP
Ippoliti, 2021 [33]	CyberRwanda	Rwanda	Web‐based and smartphone application	SRH^g^ education, service navigation, access to SRH products (unspecified)	Interactive behaviour change web comic series; FAQ^h^ library; healthcare service directory; click and collect health product ordering.	Adolescents
Janssen, 2020 [34]; Janssen, 2021 [35]	HIVSmart!	South Africa and Canada	Web‐based and smartphone application	HIV self‐testing support	Instructions for completing and interpreting self testing results; HIV information; post‐testing advice.	Self‐testers in Cape Town and Montreal
Levy, 2015 [36]	Not reported	USA	Smartphone or tablet application	HIV education, service navigation	Survey to populate individualised educational content and information to support healthcare encounters.	Black MSM
Li, 2023 [37]	Not reported	China	Social media mini app	PrEP promotion, HIV and syphilis self‐testing, health monitoring	Educational content; asynchronous messaging with clinician; HIV/syphilis self‐test ordering; HIV/syphilis testing tracker.	HIV‐negative GBMSM
Lindayani, 2021 [38]	HIV Info Corner	Indonesia	Smartphone or tablet application	HIV education	Risk assessment; multimedia educational content; in app and email advice messages.	Adolescents
Liu, 2024 [39]	PrEPmate and DOT Diary	USA	Interactive SMS^i^ and smartphone application	PrEP management and adherence	PrEPmate: Two‐way messaging between user and HCW; medication and appointment reminders; video peer testimonials. DOTDiary: Automated DOT^j^, information about PrEP protection levels, PrEP pill‐taking reminders, a sexual behaviour diary, and a PrEP dosing and sexual activity calendar; sexual risk calculator; gamification.	HIV‐negative, Spanish‐speaking MSM and English‐ and Spanish‐speaking transgender women
Mauka, 2021 [40]	Jichunge	Tanzania	Smartphone or tablet application	PrEP management and adherence, HIV education	Pill‐taking diary; feedback on PrEP adherence; timed reminders; two way messaging between user and peer educators or HCP^k^; educational content; gamification and quizzes.	MSM and female sex workers
Nadarzynski, 2021 [41]	PAT	UK	Chatbot	SRH and HIV/STI education	AI responses to simple queries regarding SRH and HIV/STI prevention.	Not specified
Rael, 2023 [42]	SMARTtest	USA	Smartphone or tablet application	HIV and syphilis self‐testing support	Instructions for completing and interpreting self testing results; information on healthcare services; image capture and interpretation.	Not specified
Sales, 2019 [43]	SELP (Syphilis [H]ELP)	Brazil	Smartphone or tablet application	Congenital syphilis prevention; partner notification and service navigation	Multimedia educational content; health service locator; appointment scheduling; partner notification.	Men who have sex with women
Schaaf, 2024 [44]	Communication and Tracing App HIV (COMTRAC‐HIV)	Germany	Smartphone or tablet application	PrEP (and ART) management and adherence	Symptom diary; Medications; Video‐call and messaging, including medication prescription.	PrEP and ART^l^ users
Schnoor, 2024 [45]	Directlab Online	The Netherlands	Website or web‐based application	HIV/STI self‐testing	Triage; recommendation of tests; self‐test delivery; pathology collection appointment scheduling; results; notification of deviant results to HCP.	Not specified
Sharma, 2022 [46]	Be in the Know Zambia (BITKZ)	Zambia	Website or web‐based application	SRH education	Interactive behaviour change web comic series; visual guides; sharable SRH information; FAQs; quizzes; gamification.	Adolescents and youths
Sun, 2020 [47]	Trans Women Connected	USA	Smartphone or tablet application	PrEP promotion, service navigation	Vision board; educational activity; interactive LGBTQ^m^‐friendly service map.	Transgender women
Velloza, 2024 [48]	DOT Diary	USA	Smartphone or tablet application	PrEP management and adherence	Automated DOT, information about PrEP protection levels, PrEP pill‐taking reminders, a sexual behaviour diary, and a PrEP dosing and sexual activity calendar.	Young PrEP eligible MSM
Visser, 2020 [49]	iloveLife. mobi	South Africa	Website or web‐based application	SRH education	Interactive multimedia educational content (i.e., short articles, audio drama, quizzes, self‐assessments, discussion forums).; gamification.	Adolescents and youths
Widman, 2016 [50]	ProjectHeartForGirls. com	USA	Website or web‐based application	Promotion of condom use	Interactive multimedia educational content (e.g., quizzes, Q&A^n^ sheets, animated videos).	Ethnically diverse adolescent girls
Wu, 2019 [51]	Rainbow Clinic	China	Platform within dating app	Service navigation, mental health support and peer support	‘Gay‐friendly’ physician finder; online psychological consultation; in‐person appointment scheduling; peer support.	MSM
Ybarra, 2019 [52]	Guy2Guy	USA	Interactive SMS	HIV/STI education, Promotion of condom use, HIV testing promotion, peer support	Educational content delivered via automated messaging; quizzes; on‐demand advice; peer support; gamification.	Gay, bisexual, and/or queer adolescent boys

^a^ Human immunodeficiency virus.

^b^ Sexually transmitted infections.

^c^ Gay, bisexual and other men who have sex with men.

^d^ HIV pre‐exposure prophylaxis.

^e^ Men who have sex with men.

^f^ Lesbian, gay, bisexual and transgender.

^g^ Sexual and reproductive health.

^h^ Frequently asked questions.

^i^ Short messaging service.

^j^ Directly observed therapy.

^k^ Health care provider.

^l^ Anti‐retroviral therapy.

^m^ Lesbian, gay, bisexual, transgender and queer.

^n^ Question and answers.

### 3.3 Domains of User Input and Methods Used to Explore Them

A summary of the methods used, and the domains of user input sought in the development and refinement of DSH tools is provided in Table 4. In roughly two‐thirds of studies (*n* = 23), qualitative methods such as interviews, focus groups and free‐text responses to surveys were complemented by quantitative measures such as self‐report measures and engagement analytics. Further description of the range of methods used to explore various domains of user input is provided in the text that follows.

**Table 4:** Methods and domains of user input sought in digital sexual health projects (*n* = 34).

First author, year	Study design	Methods used and number of user participants	Domains of user input sought						
			Formative a	Acceptability b	Accessibility c	Usability d	Satisfaction e	Engagement f	Impact g
Adeagbo, 2021 [16]	Mixed methods	Survey (*n* = 232); semi‐structured interviews (*n* = 20)	Yes	Yes		Yes	Yes		Yes
Anderson, 2022 [17]	Qualitative only	Focus groups (*n* = 44)	Yes		Yes	Yes	Yes		
Bailey, 2016 [18]	Mixed methods	Focus groups (*n* = 16); interviews (*n* = 18); outcome questionnaire (*n* = 84)	Yes			Yes	Yes		Yes
Biello, 2021 [19]	Mixed methods	Outcome questionnaire (*n* = 71); interviews (*n* = 37)	Yes	Yes	Yes	Yes	Yes	Yes	Yes
Braddock, 2023 [20]	Qualitative only	User testing (*n* = 13)	Yes			Yes			
Chandler, 2020 [21]; Chandler, 2023 [22]	Mixed methods	Focus groups (*n*= 23); SUS^h^, questionnaire and semi‐structured interview (*n* = 8)	Yes	Yes	Yes	Yes	Yes	Yes	
Cheah, 2024 [23]	Mixed methods	SUS, outcome questionnaire, think aloud interviews and semi‐structured interviews (*n* = 14)	Yes	Yes	Yes	Yes	Yes		
Cordova, 2018 [24]	Mixed methods	Session evaluation form, CSQ^i^, focus groups and interviews (*n* = 30)	Yes	Yes	Yes	Yes	Yes		Yes
Danielson, 2016 [25]	Mixed methods	Think aloud interviews and WAMMI^j^ (*n* = 18)	Yes			Yes		Yes	
Engen, 2021 [26]; Baraitser, 2023 [27]	Mixed methods	Semi‐structured interviews (*n* = 15; *n* = 10)	Yes	Yes	Yes	Yes	Yes		Yes
Fitzpatrick, 2023 [28]	Mixed methods	SUS and patient‐reported experience measures (*n* = 81) with open‐ended responses (*n* = 74)	Yes	Yes		Yes	Yes		Yes
Gilbert, 2017 [29]	Qualitative only	Think aloud interviews (*n* = 15), semi‐structured interviews (*n* = 13)	Yes	Yes	Yes	Yes	Yes		Yes
Goldenberg, 2015 [30]	Qualitative only	Focus groups (*n* = 34)	Yes	Yes	Yes	Yes			
Greene, 2016 [31]	Mixed methods	Abbreviated Acceptability Rating Profile and self‐report outcome measures (Sexual Health and HIV Risk Behaviours, HIV Knowledge Questionnaire, Condom Use Errors and Problems Questionnaire (*n* = 343); open ended survey questions (*n* = 168)		Yes		Yes	Yes		Yes
Hughes, 2021 [32]	Mixed methods	Semi‐structured interviews and chart review (*n* = 31)		Yes		Yes	Yes	Yes	
Ippoliti, 2021 [33]	Qualitative only	Prototyping, in‐depth interviews, focus groups and engagement data (*n* = unspecified)	Yes	Yes	Yes	Yes		Yes	
Janssen, 2020 [31]; Janssen, 2021 [35]	Qualitative only	Semi‐structured interviews (*n* = 34) and observations.		Yes	Yes	Yes	Yes		Yes
Levy, 2015 [36]	Mixed methods	Survey (*n* = 93) with open ended question (*n* = 42)	Yes	Yes					Yes
Li, 2023 [37]	Mixed methods	In‐depth interviews (*n* = 18), self‐reported engagement and SUS (*n* = 43)	Yes	Yes		Yes	Yes	Yes	Yes
Lindayani, 2021 [38]	Mixed methods	Think aloud interviews, SUS, Intrinsic Motivation Inventory and global rating (*n* = 15)				Yes	Yes		
Liu, 2024 [39]	Mixed methods	Focus groups (*n*= 15), SUS, CSQ, PrEP Adherence Self‐Efficacy Scale and app engagement data (*n* = 21), and brief qualitative interview (*n* = 17)	Yes	Yes	Yes	Yes	Yes	Yes	Yes
Mauka, 2021 [40]	Mixed methods	Pre‐ and post‐pilot focus groups and engagement data (*n* = 20)	Yes	Yes		Yes	Yes	Yes	Yes
Nadarzynski, 2021 [41]	Qualitative only	Semi‐structured interviews (*n* = 40)		Yes		Yes	Yes		Yes
Rael, 2023 [42]	Mixed methods	Computer assisted self interview of user experience and outcomes and in‐depth interviews (*n* = 11)		Yes		Yes	Yes	Yes	Yes
Sales, 2019 [43]	Mixed methods	Usability questionnaire and interview (*n*= 8)		Yes	Yes	Yes	Yes		Yes
Schaaf, 2024 [44]	Qualitative only	Focus groups and Think aloud interviews (*n* = 7)			Yes	Yes	Yes		
Schnoor, 2024 [45]	Qualitative only	Focus groups (*n* = 19 total; *n* = 9 HIV/STI related group)		Yes	Yes	Yes			
Sharma, 2022 [47]	Mixed methods	Pre‐ and post‐pilot self‐report outcome measures (*n* = 749 in intervention group), engagement data (*n* = 601), in‐depth interviews (*n* = 59)	Yes		Yes		Yes	Yes	Yes
Sun, 2020 [47]	Mixed methods	Think aloud interviews, ratings of difficulty, appearance, perceived usefulness and educational value, and open questions (*n* = 16)	Yes	Yes		Yes	Yes		Yes
Velloza, 2024 [48]	Qualitative only	Semi‐structured, in‐depth interviews (*n* = 24)	Yes	Yes	Yes	Yes	Yes		Yes
Visser, 2020 [49]	Mixed methods	Knowledge, attitudes, practices and behaviours survey (*n*= 1882), focus groups (*n* = 68), interviews (*n* = 175)				Yes	Yes	Yes	Yes
Widman, 2016 [50]	Qualitative only	Think aloud interviews (*n* = 6)	Yes		Yes	Yes	Yes		
Wu, 2019 [51]	Mixed methods	Focus groups and questionnaire (*n* = 34)	Yes	Yes		Yes			Yes
Ybarra, 2019 [52]	Mixed methods	Post‐intervention survey (*n* = 132), asynchronous focus groups (*n* = 45)	Yes	Yes			Yes		Yes
Number of projects seeking this domain of input			24	25	17	31	27	11	22

^a^ Formative contribution to design and scoping.

^b^ Willingness or preference to use.

^c^ Barriers to use.

^d^ Ease of use.

^e^ Satisfaction or experience of use.

^f^ Spontaneous engagement with tool or specific features.

^g^ Perceived or actual impact.

^h^ System Usability Scale.

^i^ Client Satisfaction Questionnaire.

^j^ Website Analysis and MeasureMent Inventory.

#### 3.3.1 Formative Input

Twenty‐four of thirty‐four projects reported seeking user input in the development of the prototype or tools they evaluated. This included conducting interviews or focus groups with end users on their sexual health needs or consulting with advisory groups. A further seven studies did not report on this aspect of development, and three reported tool development by subject matter experts without user input.

Those studies that sought formative user input created tools that were more closely aligned with the suggestions made by users providing feedback at later stages of development in other tools. For example, a crowdsourcing call reported in Wu et al.'s study [51] resulted in the development of a physician finder tool that men who have sex with men could use to find gay‐friendly doctors. Similar suggestions were made by focus group participants in the formative stages of the SavvyHER application, which incorporated a feature identifying local HIV testing locations: ‘… something where you could get a directory of… Female Black doctors in your area. I think that would be very, very helpful because I have no idea of any Black women doctors in Atlanta’. [Focus group participant] [18]. Similar requests for information about the location of sexual healthcare providers were made by teenage girls in later stages of tool development in Widman et al.‘s [50] and Levy et al.'s [36] studies, but it was unclear whether this suggestion was taken up by the developers.

#### 3.3.2 Acceptability

The acceptability of tools to users was explored in twenty‐five of the thirty‐four projects. Quantitative measures of this domain included a mix of those designed for the specific study [22, 28, 47, 52] and standardised measures. The latter included the *Abbreviated Acceptability Rating Profile* [24, 39]. Qualitative feedback indicated features that impacted the acceptability of tools, such as anonymity, privacy, convenience and appearance.

Black women in Chandler et al.'s [22] study commented on the representativeness of a tool designed with formative input. In contrast, a self‐testing tool that had not had formative input from transgender users received negative feedback on its acceptability.

Put the [transgender pride] flag up in a corner… and maybe a girl with long hair, and a hand with long fingernails. Tonterias [silly things] like that… that'll make you laugh and giggle…. You don't want it to seem–… I'm… doing an institutional thing–I feel like I got the Feds hearing me.

[Interview participant] [42]

Participants also speculated about the acceptability of specific application features to other users: ‘It's a matter of privacy with the reminders. I personally don't care if people see my phone when the notification is going to come up. But some people don't want that kind of stuff visible’ [Focus group participant] [30].

Qualitative descriptions of acceptability also compared digital tools to in person care: ‘I understand that as being the trade‐off. For like, the ease and simplicity, vs. like, having that like, specific person to contact and like, them being like, readily available’ [Interview participant] [32].

#### 3.3.3 Accessibility

User input relevant to accessibility was reported in only half of included projects, and most often in general terms rather than intentional consideration of users with special needs. No quantitative measures were identified capturing feedback on accessibility. However, qualitative feedback from users reflected potential barriers to use for some users.

Mixed feedback from participants on comprehension of text in some studies [18, 28] suggests consideration of accessibility for people with low literacy or English language proficiency. For example, Biello et al.'s [19] study of a tool for self‐testing indicated the instructions provided were not accessible for some users: ‘…what am I supposed to—I didn't know why I got confused honestly, but… I just… kept putting them off, because I was, like… I don't want to do this wrong’ [Interview participant]. Similarly, user input suggesting or appreciating the use of voiceovers, video, and visual guides are relevant to the accessibility of text‐based content [20, 23, 32, 42, 45, 49].

User input relevant to accessibility to people with visual impairments included the size of ‘buttons’ [43], contrast of text [16], concern about working on a small screen [25], modification of visual presentation [43], and sounds [38, 47].

#### 3.3.4 Usability

Use of methods to assess the usability (also referred to as ‘ease‐of use’) of tools was the most frequently reported (*n* = 31) domain of user feedback. The most frequently used standardised measure, of any user feedback domain, was the *System Usability Scale* which was used in six projects [22, 23, 28, 37, 38, 39] but the *Website Analysis and MeasureMent Inventory* was also used by Danielson et al. [25]. A commonly used (*n* = 7) qualitative method to assess usability was ‘think aloud’ interviews, which enabled researchers to identify usability problems while users were using the tool [23, 25, 29, 38, 44, 47, 50]. Other forms of qualitative feedback described how usability could impact user satisfaction and their future uptake: ‘… the amount of times that I had to try to take those pictures and to get them online, I thought that was one of the biggest cons’’ [Interview participant] [26].

#### 3.3.5 Satisfaction

Satisfaction (also referred to as user experience) was the next most common (*n* = 27) domain of user feedback sought by tool developers. Quantitative measures of satisfaction included a mix of those designed for the specific study [22, 28, 47, 52] and standardised measures. The latter included the *Client Satisfaction Questionnaire* [24, 39], and the *Intrinsic Motivation Inventory* [38]. Six studies measured satisfaction by asking users if they would recommend the tool to a friend [23, 24, 28, 40, 47, 52]. Qualitative feedback allowed participants to describe factors impacting their satisfaction that might not be captured in quantitative data: ‘I ‘ain't want to say that's aggressive either, but the way she [person in educational video] talking sound aggressive’ [Interview participant] [25].

#### 3.3.6 Engagement

Given that not all included projects had progressed to user testing, data relating to whether participants spontaneously used tools was reported in less than a third of the tools evaluated (*n* = 11). In studies that involved a pilot period of user testing, quantitative measure of engagement were more frequently extracted from the tool [33, 39, 40, 47] than self‐reported by participants [37]. In addition to how often and for how long they used tools, qualitative feedback on engagement described how and when they might be used.

I used the HIV self‐test kit and read some articles…. Once after I know all about this [PrEP], I don't use it often. If I suddenly forget about something [about PrEP] or not sure of something, I will go back [to the mini‐app]. Or when I am really sick or need to look up something. If others ask me [about PrEP], I would just refer them to the mini‐app, or share some of the articles to them.

[Interview participant] [37]

Qualitative feedback also captured unexpected engagement with tools that might not have been reflected in quantitative measures of engagement. For example, an interview participant without internet access in Mauka et al.'s [40] study was prompted to take her PrEP dose by seeing the application logo on her phone: ‘I have never received the message but, since I like to use the phone, once I see the app's logo, I remember to take medicine’.

#### 3.3.7 Impact

Users’ feedback on the potential or actual impact of tools on their sexual health knowledge or behaviours was commonly reported (*n* = 22). Self‐report outcome questionnaires were used in several studies to assess impacts on sexual health knowledge and engagement with prevention (e.g., condom use, PrEP use) and HIV/STI testing. These questionnaires were often designed for the study [16, 18, 23, 42, 47, 49, 52]. Standardised measures of impact on knowledge and behaviour, such as the *HIV Knowledge Questionnaire, Sexual Health and HIV Risk Behaviours*, and the *Condom Use Errors and Problems Questionnaire* [31], and confidence, such as the *PrEP Adherence Self‐Efficacy Scale* [39], were used rarely. Qualitative methods captured impacts on similar outcomes to those measured in self‐report measures, providing more nuanced description of changes that occurred: ‘The iloveLife.mobi site gave me knowledge to confront my fear of knowing my HIV status. I have been to the clinic and tested for HIV’ [Survey participant] [49].

### 3.4 Reported Incorporation of User Input

User centred research and design approaches cited in studies included ‘human centred design’ [26, 44], ‘codesign’ [28, 33], ‘human computer interaction person‐based’ [16], ‘community‐engaged’ [21], ‘community based participatory research’ [24], ‘user centred design’ [39], participatory design of interaction [43], ‘user‐driven’ [47], and ‘crowdsourcing’ [51]. However, references to such approaches were not consistently supported by evidence of changes to tools based on user input. Suggestions for changes or additional features were reported in many studies (*n* = 25). These suggestions, sometimes reflected a mismatch between the needs of users, as perceived by tool developers, and the reported needs of users which might be met by different technological solutions, information content or tool features. For example, a survey participant in Greene et al.'s [31] evaluation of a website promoting condom use suggested they: ‘take into consideration the new PrEP [HIV pre‐exposure prophylaxis] medicine and also give realistic happenings without condom use’. Most studies (*n* = 20) failed to report changes made or planned to tools in response to these suggestions.

## 4 Discussion

### 4.1 Summary of Evidence

This scoping review of published qualitative and mixed methods studies of DSH tools sought to describe the approaches taken to seeking and incorporating user input. Studies were reviewed in relation to the domains of input sought from potential users, and the degree to which user contributions were incorporated into the development and refinement of tools.

In relation to our first aim – identifying the types of user input sought ‐ projects included in this review most often sought input on usability. Formative input and input regarding the acceptability of tools and their features was not sought in a third of projects. Crucially, few studies reported asking users whether they saw a need for the tool, in the first place, or what type of tool would best meet their needs. Only half of projects reported findings relevant to accessibility, and often without purposefully seeking this input in their research design. Usability may be irrelevant if a tool is not useful, appealing or accessible to the population it is designed to target.

In relation to our second aim, reported incorporation of user input was similarly variable. In part, this may reflect the earlier developmental stage of some tools at the time of publication. However, where user input was clearly incorporated into iterative tool development, subsequent feedback from users reflected a high degree of satisfaction, suggesting potential for further engagement and impact, as proposed in our theory of change (Figure 1). This assertion is supported by Veronese et al.'s [1] recent review of digital interventions to improve HIV testing that found tools incorporating user input were more impactful. While the engagement of target populations in the development of complex health interventions is increasingly mandated in health policy and research practice [53], evaluations linking processes of seeking user input with health intervention outcomes are limited [9, 10]. Future research in the DSH space might benefit from evaluating theories of change [15], such as the one we have proposed in Figure 1. Moreover, combining explanatory models from both the digital (e.g., unified theory of acceptance [54], privacy calculus [55]) and experience‐based co‐design (EBCD) [56] literature (e.g., theoretical explanatory model of change for co‐design and co‐production in healthcare improvement [57]) may provide insight into mechanisms of change. In comparing approaches from technology and community‐focussed research, Chen et al. [8] argue that integrating human‐centred design (HCD) and community‐based participatory research (CBPR) approaches may result in improved impact of public health interventions. While both are ‘people‐centred’ approaches focussed on iterative co‐creation of solutions, each approach has unique and complementary strengths that may be leveraged in the design of DSH tools. Aspects of EBCD, HCD, CBPR, and crowdsourcing approaches will be discussed in relation to the review findings in the following guidance for developers of DSH tools, while acknowledging that other approaches to seeking user input may be equally relevant and valid.

### 4.2 Suggested Guidance for Developers of Digital Sexual Health Tools

#### 4.2.1 Involve End Users Early

Seeking input from end users earlier in the development process, allowed the developers of DSH tools to establish the need for a tool, and incorporate user suggestions into their tools, such as the type of tool and its features. In contrast, suggestions made at later stages of development were not consistently reported as being incorporated into tools. Moreover, authors who had only included clinician perspectives in formative work reflected on the limitations of this approach.

The app's design solution used in this study was initially based on the view of HIV experts. Subsequently involving patients is essential, as they identify needs not mentioned by the experts. We therefore recommend early consideration of clinical perspectives and patient needs and discussing them with experts [44].

The findings of our study suggest that the choice of method for seeking input is less important than the choice to prioritise users’ needs from the outset of tool development. Consistent with the information gathering stage of experience‐based co‐design [56], interviews, focus groups and surveys have been successfully used in the early stages of online sexual health services to gain valuable insights into perceptions and likely uptake [58]. Beyond traditionally used methods, methods from other fields offer inspiration for developers. Crowdsourcing methods from the field of behavioural economics have been used successfully to generate ideas from target populations [50, 59]. In the field of technology, HCD is described by Chen et al. [8] as a highly a creative approach which may support the generation of novel technological solutions rather than use of existing technologies to meet a healthcare need identified through participatory research.

#### 4.2.2 Engage Your Target Population, Including ‘Extreme Users’

While many included studies reported use of person‐centred approaches, users engaged in the development of tools were not always representative of the populations who might most benefit from tools. For example, participants in some studies of tools targeting a general population were mostly female [26, 41], white [41] or born in the country of study [29]. Some tools targeting adolescent and youth populations excluded participants under 18 years [16, 50]. Some included studies seeking user input on tools promoting PrEP recruited almost exclusively male [28, 44] participants and those over 30 years of age [44].

DSH tools are often promoted as a means of improving access to sexual health information and services for populations experiencing barriers to in person care. However, exclusion of users with access needs (e.g., people with limited English language proficiency or low literacy, people with disabilities) from formative research may serve to deepen health inequalities by providing convenient access for those already able to access information and services in other ways. Chen et al. [8] recommend researchers adopt the HCD approach of working ‘extreme’ users to ensure public health interventions are accessible to a wider audience. However, HCD approaches tend to focus on scalability rather than the localised approach of participatory research which may be better suited to populations with specific needs. In relation to DSH tools, developers should seek to ensure their target audience is represented in those they seek input from as this has been correlated with improve impact on outcomes [1]. Moreover, to ensure accessibility, behavioural science approaches, such as the behaviour change wheel, may be helpful in systematically identifying and addressing barriers to, and facilitators of, use [60].

#### 4.2.3 Create Opportunities for Unexpected Input

While this review excluded studies where only quantitative user input was sought, comparison of the quantitative and qualitative data presented in mixed methods studies suggest that the inclusion of qualitative data in formative evaluations of DSH tools is essential to capture more nuanced and open input. This was particularly true of studies which employed interview or focus group methods and, to a lesser degree, open text responses to questionnaires. Where quantitative data was collected regarding acceptability, satisfaction or engagement, qualitative data provided explanation of quantitative findings. Given the mixed findings of several reviews of quantitative studies of DSH tools it may be time to recognise the value of qualitative data in tool development [1, 2, 4].

#### 4.2.4 Involve Users More Than Once

Projects that included users throughout the development process were more likely to report making changes to their planned approach and positive feedback from users on prototypes or final products. Chen et al. [8] suggest conducting rapid and repeated rounds of prototyping towards tangible outputs that users can test. This contrasts with other forms of qualitative and participatory research which can be slow in generating outputs.

#### 4.2.5 Close the Feedback Loop

A notable gap in reporting was changes made to tools in response to user input. An essential aspect of co‐design processes is transparency in how input was used, as prioritised in the relational approach of participatory research [8, 57]. Reporting in research publications demonstrates commitment to responding to user input. In addition to reporting how feedback was used, the revised *Guidance for Reporting Involvement of Patients and the Public* provides a benchmark for reporting [61].

### 4.3 Strength and Limitations

This review undertook a rigorous process of searching, screening, data extraction and synthesis of peer reviewed studies of DSH tools. The diverse disciplinary and regional perspectives amongst coauthors further supported a holistic approach to data analysis. While several literature reviews of DSH applications have been published, this is the first to focus on the methods used and domains of input sought by researchers.

The review is limited in that we did not search in languages other than English, preprints or the proceedings of conferences, or other grey literature. Given the fast moving and competitive digital field, it seems likely that many more applications are developed through market, rather than health, research. Moreover, as we were using published research from various stages of tool development, our data extraction and analysis may have omitted aspects of projects not, or not yet, reported by authors. Similarly, our choice to exclude purely formative research may have limited our ability to assess this domain, if these studies were not referenced in later publications. Limitations in the reporting of included studies further limited the scope of our analysis. For example, researchers did not make explicit commercial interests that may have influenced the reporting of negative feedback or the limitations in accessibility of tools due to cost. Qualitative findings in included studies also tended to be reported as themes rather than exploring relationships between factors and outcomes.

A significant limitation of the review is the omission of Arksey and O'Malley's [11] suggested sixth stage of stakeholder consultation, which, due to resourcing limitations, we were unable to undertake. Seeking the views of potential users of DSH tools would have added depth and nuance to our interpretation of the findings. While we adhered to established scoping review methodology and the Preferred Reporting Items for Systematic reviews and Meta‐Analyses extension for Scoping Reviews (PRISMA‐ScR) [54] no protocol was published and, thus, our methods not subjected to peer review and revision.

## 5 Conclusions

This review found inconsistent user input into the development of DSH tools which may, in part, explain their mixed effectiveness in improving sexual health outcomes, as reported in previous systematic reviews [1, 2, 3, 4]. If DSH tools are to achieve their promise, we must move beyond evaluating the functionality of design solutions and meaningfully engage users as partners in co‐design. Future research should seek to ensure fidelity to established principles of co‐design and build the evidence base for the impact of these approaches on outcomes.

## Author Contributions

**Alicia King:** conceptualisation, methodology, formal analysis, investigation, validation, data curation, writing – original draft, visualisation, project administration. **Ethan Cardwell:** conceptualisation, methodology, formal analysis, investigation, data curation, writing – review and editing. **Eric Chow:** funding acquisition, writing – review and editing, supervision. **Paul Flowers:** conceptualisation, methodology, writing – review and editing. **Mark Gilbert:** methodology, writing – review and editing. **Kath Albury:** conceptualisation, methodology, validation, writing – review and editing, supervision.

## Ethics Statement

The authors have nothing to report.

## Consent

The authors have nothing to report.

## Conflicts of Interest

The authors declare no conflicts of interest.

## Supporting information

UserInputinDSH_Supplement_1.

UserInputinDSH_Supplement_2.

UserInputinDSH_Supplement_3.

UserInputinDSH_Supplement_4.

## Data Availability

Data sharing is not applicable to this article as no new data were created or analysed in this study.
