# Supplementary material for: User Input in the Development of Digital Sexual Health Tools: A Scoping Review and Guidance for Tool Developers
Source: Health Expect. 2025 Jul 28;28(4):e70360. doi: 10.1111/hex.70360 (PMC12301633; doi:10.1111/hex.70360)
Supplement: Supplementary file 1 — UserInputinDSH_Supplement_1. [file HEX-28-e70360-s001.docx]

1. Digital health.mp. or exp Digital Health/

2. applications.mp. or exp Mobile Applications/

3. exp User-Computer Interface/

4. 1 or 2 or 3

5. Sexually Transmitted Diseases.mp. or exp Sexually Transmitted Diseases/

6. Sexually Transmitted Infections.mp. [mp=title, book title, abstract, original title, name of substance word, subject heading word, floating sub-heading word, keyword heading word, organism supplementary concept word, protocol supplementary concept word, rare disease supplementary concept word, unique identifier, synonyms, population supplementary concept word, anatomy supplementary concept word]

7. Sexual Transmissible Infections.mp. [mp=title, book title, abstract, original title, name of substance word, subject heading word, floating sub-heading word, keyword heading word, organism supplementary concept word, protocol supplementary concept word, rare disease supplementary concept word, unique identifier, synonyms, population supplementary concept word, anatomy supplementary concept word]

8. Chlamydia.mp. or exp Chlamydia/

9. Gonorrhea.mp. or exp Gonorrhea/

10. Gonorrhoea.mp. [mp=title, book title, abstract, original title, name of substance word, subject heading word, floating sub-heading word, keyword heading word, organism supplementary concept word, protocol supplementary concept word, rare disease supplementary concept word, unique identifier, synonyms, population supplementary concept word, anatomy supplementary concept word]

11. exp HIV/ or HIV.mp.

12. syphilis.mp. or exp Syphilis/

13. herpes.mp. or exp Herpes Simplex/

14. HPV.mp. or exp Papillomavirus Infections/

15. 5 or 6 or 7 or 8 or 9 or 10 or 11 or 12 or 13 or 14

16. Sexual health.mp. or exp Sexual Health/

17. Sex education.mp. or exp Sex Education/

18. exp Sexual Behavior/ or sexual behav*.mp.

19. exp "Patient Acceptance of Health Care"/ or Healthcare engagement.mp.

20. exp Primary Prevention/ or exp Secondary Prevention/ or prevention.mp.

21. exp Condoms/ or condom*.mp.

22. self-testing.mp. or exp Self-Testing/

23. HIV testing.mp. or exp HIV Testing/

24. exp Pre-Exposure Prophylaxis/ or PrEP.mp. or exp Anti-HIV Agents/

25. exp Vaccination/ or vaccinat*.mp.

26. Qualitative research.mp. or exp Qualitative Research/

27. User experience.mp. [mp=title, book title, abstract, original title, name of substance word, subject heading word, floating sub-heading word, keyword heading word, organism supplementary concept word, protocol supplementary concept word, rare disease supplementary concept word, unique identifier, synonyms, population supplementary concept word, anatomy supplementary concept word]

28. Patient Participation.mp. or exp Patient Participation/

29. Mixed method*.mp. [mp=title, book title, abstract, original title, name of substance word, subject heading word, floating sub-heading word, keyword heading word, organism supplementary concept word, protocol supplementary concept word, rare disease supplementary concept word, unique identifier, synonyms, population supplementary concept word, anatomy supplementary concept word]

30. Survey.mp. or exp "Surveys and Questionnaires"/

31. (Co-design or codesign).mp. [mp=title, book title, abstract, original title, name of substance word, subject heading word, floating sub-heading word, keyword heading word, organism supplementary concept word, protocol supplementary concept word, rare disease supplementary concept word, unique identifier, synonyms, population supplementary concept word, anatomy supplementary concept word]

32. ("human centered design" or "human centred design" or "human-centered design" or "human-centred design").mp. [mp=title, book title, abstract, original title, name of substance word, subject heading word, floating sub-heading word, keyword heading word, organism supplementary concept word, protocol supplementary concept word, rare disease supplementary concept word, unique identifier, synonyms, population supplementary concept word, anatomy supplementary concept word]

33. Universal design.mp. or exp Universal Design/

34. human computer interaction.mp.

35. healthcare seeking.mp.

36. exp Community-Based Participatory Research/ or participatory research.mp.

37. 16 or 17 or 18 or 19 or 20 or 21 or 22 or 23 or 24 or 25 or 35

38. 26 or 27 or 28 or 29 or 30 or 31 or 32 or 33 or 34 or 36

39. 4 and 15 and 37 and 38
