# Supplementary material for: User Input in the Development of Digital Sexual Health Tools: A Scoping Review and Guidance for Tool Developers
Source: Health Expect. 2025 Jul 28;28(4):e70360. doi: 10.1111/hex.70360 (PMC12301633; doi:10.1111/hex.70360)
Supplement: Supplementary file 2 — UserInputinDSH_Supplement_2. [file HEX-28-e70360-s003.docx]

**Database: APA PsycInfo <1806 to May Week 5 2024>**
**Search Strategy:**
**1**  exp Telemedicine/ or Digital health.mp. or exp Digital Interventions/ (18175)
**2**  exp Mobile Applications/ or exp Computer Applications/ or applications.mp. (166201)
**3**  1 or 2 (168436)
**4**  Sexually Transmitted Diseases.mp. or exp Sexually Transmitted Diseases/ (55038)
**5**  Sexually Transmitted Infections.mp. (4102)
**6**  Sexual Transmissible Infections.mp. (0)
**7**  chlamydia.mp. (1082)
**8**  exp Gonorrhea/ or Gonorrhea.mp. (740)
**9**  Gonorrhoea.mp. (191)
**10**  exp HIV/ or HIV.mp. (66243)
**11**  exp Syphilis/ or syphilis.mp. (2037)
**12**  exp Herpes Simplex/ or herpes.mp. or exp Herpes Genitalis/ (2595)
**13**  HPV.mp. or exp Human Papillomavirus/ (2466)
**14**  4 or 5 or 6 or 7 or 8 or 9 or 10 or 11 or 12 or 13 (76088)
**15**  exp Sexual Health/ or Sexual health.mp. (8937)
**16**  sex education.mp. or exp Sex Education/ (9437)
**17**  sexual behav*.mp. (49619)
**18**  healthcare engagement.mp. (61)
**19**  exp Health Care Seeking Behavior/ or healthcare seeking behavior.mp. (10624)
**20**  prevention.mp. or exp Prevention/ (180190)
**21**  exp Condoms/ or condom.mp. (9476)
**22**  self-testing.mp. (462)
**23**  HIV testing.mp. or exp HIV Testing/ (5356)
**24**  PrEP.mp. or exp Pre-Exposure Prophylaxis/ (2621)
**25**  exp Vaccination/ or vaccination.mp. (7924)
**26**  15 or 16 or 17 or 18 or 19 or 20 or 21 or 22 or 23 or 24 or 25 (254773)
**27**  Qualitative research.mp. or exp Qualitative Methods/ (65463)
**28**  exp Product Design/ or user experience.mp. (11654)
**29**  patient participation.mp. or exp Client Participation/ (8812)
**30**  exp Mixed Methods Research/ or mixed method.mp. (11166)
**31**  exp Online Surveys/ or exp Consumer Surveys/ or exp Surveys/ or survey.mp. (361704)
**32**  codesign.mp. (164)
**33**  human centered design.mp. (276)
**34**  human centred design.mp. (88)
**35**  universal design.mp. (891)
**36**  human computer interaction.mp. or exp Human Computer Interaction/ (28262)
**37**  exp Action Research/ or participatory research.mp. (8747)
**38**  27 or 28 or 29 or 30 or 31 or 32 or 33 or 34 or 35 or 36 or 37 (478674)
**39**  3 and 14 and 26 and 38 (135)
