# Supplementary material for: User Input in the Development of Digital Sexual Health Tools: A Scoping Review and Guidance for Tool Developers
Source: Health Expect. 2025 Jul 28;28(4):e70360. doi: 10.1111/hex.70360 (PMC12301633; doi:10.1111/hex.70360)
Supplement: Supplementary file 3 — UserInputinDSH_Supplement_3. [file HEX-28-e70360-s004.docx]

**1. ((ALL=(digital health)) OR ALL=(telemedicine)) OR ALL=(application)**

**2. ((((((((ALL=(sexually transmitted diseases)) OR ALL=(sexually transmitted infections)) OR ALL=(sexually transmissible infections)) OR ALL=(chlamydia)) OR ALL=(gonorrh*)) OR ALL=(syphilis)) OR ALL=(HIV)) OR ALL=(HPV)) OR ALL=(herpes)**

**3. ((((((((((ALL=(sexual health)) OR ALL=(sex education)) OR ALL=(sexual behav*)) OR ALL=(healthcare engagement)) OR ALL=(healthcare seeking)) OR ALL=(prevention)) OR ALL=(condom*)) OR ALL=(self-testing)) OR ALL=(HIV testing)) OR ALL=(PrEP)) OR ALL=(vaccinat*)**

**4. ((((((((((ALL=(qualitative research)) OR ALL=(user experience)) OR ALL=(patient participation)) OR ALL=(mixed method*)) OR ALL=(survey)) OR ALL=(codesign)) OR ALL=(human centered design)) OR ALL=(human centred design)) OR ALL=(universal design)) OR ALL=(human computer interaction)) OR ALL=(participatory research)**

**5. #1 AND #2 AND #3 AND #4**
