# Supplementary material for: User Input in the Development of Digital Sexual Health Tools: A Scoping Review and Guidance for Tool Developers
Source: Health Expect. 2025 Jul 28;28(4):e70360. doi: 10.1111/hex.70360 (PMC12301633; doi:10.1111/hex.70360)
Supplement: Supplementary file 4 — UserInputinDSH_Supplement_4. [file HEX-28-e70360-s002.docx]

| Area | Field |
| --- | --- |
| Paper details | First author |
|  | Year of publication |
|  | Title of paper |
| Setting details | Country or countries in which research was conducted |
|  | Additional research setting details (e.g., region or state, urban or rural, school) |
| Tool details | Name of tool |
|  | Description of tool design process |
|  | Type of tool (e.g., website, chatbot, mobile app) |
|  | Purpose of tool (e.g., HIV prevention, SRH education) |
|  | Description of tool |
|  | Target gender |
|  | Target age group |
|  | Target ethnicity or language group |
|  | Target sexual orientation or occupation (e.g., MSM, sex workers) |
|  | Additional sexual health related target populations (e.g., HIV negative) |
| Research details | Research aim/s |
|  | Gender or sex related inclusion/exclusion criteria |
|  | Age related inclusion/exclusion criteria |
|  | Geographic, ethnic or language related inclusion/exclusion criteria |
|  | Sexual orientation or occupation related inclusion/exclusion criteria |
|  | Other reported inclusion/exclusion criteria |
|  | Data collection methods |
|  | Number of participants by methods |
|  | Finding by research aim/s |
|  | Conclusions |
| User input | Description of user input in design phases prior to study |
|  | Description of changes or additional features suggested by participants |
|  | Description of changes planned or executed based on user input |
